# Hydrogen sulfide is expressed in the human and the rat cultured nucleus pulposus cells and suppresses apoptosis induced by hypoxia

**DOI:** 10.1371/journal.pone.0192556

**Published:** 2018-02-21

**Authors:** Haolin Sun, Longtao Qi, Shijun Wang, Xuwen Li, Chunde Li

**Affiliations:** Department of Orthopedic, Peking University First Hospital, Beijing, China; University of Louisville, UNITED STATES

## Abstract

Apoptosis plays pivotal role in the pathogenesis of degenerative disc diseases, which is the primary contributor to low back pain. Although the role of hydrogen sulfide (H_2_S) in cell apoptosis is well appreciated, the effects and mechanism that H_2_S regulates the program death of intervertebral disc cell are not yet elucidated. In this study, we utilized the nucleus pulposus (NP) from patients with lumbar disc herniation to investigate the relationship between endogenous H_2_S and NP cells apoptosis in human. Furthermore, we analyzed primary rat NP cells to study the effects of exogenous H_2_S on hypoxia induced cell apoptosis. Human NP samples were obtained from patients with lumbar disc herniation and were divided into uncontained and contained herniation groups. Using immunohistochemistry staining and sulphur-sensitive electrode, we detected the expression of cystathionine-β-synthase (CBS) and cystathionine γ-lyase (CSE), as well as the production of endogenous H_2_S in human NP. Tunel staining showed increased apoptosis in NP from herniated disc; and there was significant correlation between H_2_S generation and apoptosis in human NP. CoCl_2_ was then used to induce hypoxia in cultured primary rat NP cells. Annexin V staining indicated that exogenous NaHS attenuated hypoxia induced apoptosis in rat NP cells. Furthermore, hypoxia significantly increased the levels of multiple apoptosis associated proteins (Fas, Cytochromes C, Caspase 9 and cleaved-Caspase-3) in cells, which were eliminated by NaHS.

Our study demonstrates the presence of endogenous H_2_S in human intervertebral disc; and the endogenous H_2_S generation rate is associated with NP apoptosis in herniated disc. In vitro study showes exogenous H_2_S donor attenuates hypoxia induced apoptosis in primary rat NP cells. Thus, our work provides insights that H_2_S may have beneficial effects in treating degenerative disc diseases.

## Introduction

Intervertebral disc degeneration is frequently associated with disc herniation, low back pain and sciatica, thus leads to global burden with severe health-care and socioeconomic consequences [1, 2]. Apoptosis is a prerequisite process for the development of nucleus pulposus (NP) cells and the maintenance of tissue homeostasis. However, excess apoptosis associated with hypoxia may lead to degenerative disc diseases [3, 4], which the underlying mechanisms remain largely unknown.

Hydrogen sulfide (H_2_S) is recognized as a third gasotransmitter following nitric oxide (NO) and carbon monoxide (CO), and is physiologically present in a variety of mammalian tissues including cardiovascular system, digesting system, brain, *etc* [5]. Endogenously, H_2_S is synthesized from the precursor L-cysteine via two pyridoxal-5′-phosphate dependent enzymes, cystathionine β-synthase (CBS) and cystathionine γ-lyase (CSE). Although H_2_S was proved to be involved in multiple physiological cellular changes, including cell cycle, oxygen transduction, *etc* [6, 7], Unregulated H_2_S may contribute to the development of variety of diseases such as degenerative disease [8], cancer, inflammation and ischemia-reperfusion-induced injury [9–11].

The current knowledge regarding endogenous H_2_S in intervertebral disc is limited. Recently, Xu and colleagues showed H_2_S plays a protective role in intervertebral disc degenenration via reducing endoplasmic reticulum stress and mitochondrial injury in NP cells [12]. In this study, we focused on assesing the relationship between endogenous H_2_S production, NP cells apoptosis and intervertebral disc herniation. In addition, the effect of H_2_S on hypoxia induced NP cells apoptosis was investigated in cultured primary rat NP cells.

## Materials and methods

### Collection of human lumbar intervertebral disc

Human lumbar intervertebral disc samples were obtained during discectomy surgery from patients suffering lumbar disc herniation (between L4-S1 levels). 23 females and 17 males with average age of 49.3±7.2 were recruited from Jan 2016 to June 2016. The intervertebral disc herniation was further classified into contained (displaced disc tissue is wholly held within intact outer annulus, n = 20) or uncontained (the outer annulus is not intact and the displaced disc tissue leaks into the vertebral canal fluid, n = 20) group based on magnetic resonance imaging results and surgical findings [3]. The intervertebral disc tissues from 5 adolescent idiopathic scoliosis patients were used as control, with average age at 17.6±3.7 and pathological changes at L1-L5 levels. Before operation, surgeon presented a detailed written informed consent to the patients. All participants must signed the consent form before initiation of this study. If patients were minors, parents or guardians must signed the consent. The study was approved by the Human Subjects Institutional Review Board at Peking University First Hospital.

### Immunohistochemistry and TUNEL staining in human lumbar nucleus pulposus

NP was isolated immediately from intervertebral disc after surgery, post-fixed in 10% formalin and embedded in paraffin. Transverse sections (4um thick) were then obtained and immunohistochemistry staining was performed as previously described [13]. In brief, the sections were deparaffinized and rehydrated, endogenous peroxidase was removed using 3% hydrogen peroxide for 20 min and the non-specific antibody binding sites were blocked using 10% normal goat serum for 30 minutes at 37°C. Sections were then incubated in the mouse monoclonal anti-CBS antibody (1:100, Abova, TW) and mouse monoclonal anti-CSE antibody (1:100, Abova, TW) at 4°C overnight, followed with the polymer-HRP conjugated secondary antibody (DAKO).

Apoptosis was detected using *in situ* cell death detection kit (KeyGEN, CN) following the manufacture instructions. Shortly, after deparaffinization and rehydration, the sections were treated with proteinase K (20ug/mL) for 30 minutes then quenched in 3% hydrogen peroxide for 20min. Sections were then incubated in terminal deoxynucleotidyl transferase for 60min at 37°C in dark, followed with streptavidin-HRP reaction buffer for 30min at 37°C. Color was developed using DAB substrate and hematoxylin counterstaining was applied.

Staining pictures were obtained using high-power field microscopy (Olympus, BX41, JP), quantitative image analysis was performed by 2 investigators in a blinded manner. The total number of NP cells and CBS/CSE positive cells were counted in randomly acquired 10 nonoverlapping high-magnification imaging fields (400x) in each section and an average of CBS/CSE positive cells proportion was calculated.

### H2S production in human lumbar nucleus pulposus

Another cohort of human NP tissue was used for H_2_S measurement. The endogenous H_2_S generation was detected using sulphur-sensitive electrode based on three steps chemical reaction formula: i) *HS*^−^ + *H*^+^ ⇄ *H*2*S*; ii) 2*HS*^−^ + 2*OH*^−^ ⇉ 2*S*^2−^ + 2*H*_2_*O*; iii) *H*_2_*S* + 2*OH*^−^ ⇉ *S*^2−^ + 2*H*_2_*O* [14, 15]. In brief, NP tissues were homogenized in 50 mmol/L ice-cold potassium phosphate buffer (pH = 6.8). A reaction mixture was set up for H_2_S detection: 100 mmol/L potassium phosphate buffer (pH = 7.4), 10mmol/L L-cysteine, 2mmol/L pyridoxal 5-phosphate, and 10% (w/v) tissue homogenate. Cryovial test tubes (2 ml) were used as the center wells, and each contained 0.5 ml of 1% zinc acetate as a trapping solution along with a filter paper of 2–2.5 cm^2^ to increase the air/liquid contact surface. The reaction was performed in a 25ml Erlenmeyer flask. The flasks containing reaction mixture and the center wells were flushed with nitrogen before being sealed with a double layered parafilm. The reaction was initiated by transferring the flasks from ice to a shaking water bath at 37°C. After incubation at 37°C for 90 minutes, 0.5 ml of 50% trichloroacetic acid was added to stop the reaction. The flasks were sealed again and incubated at 37°C for 60 minutes to ensure complete trapping of the H_2_S released from the mixture. The contents of the center wells were transferred to test tubes containing 3.5 ml of water. Then, 0.5 ml of 20mmol/L N,N-dimethyl-p-phenylenediamine sulfate in 7.2mol/L HCl and 0.5 ml of 30mmol/L FeCl_3_ in 1.2mol/L HCl were added. The absorption rate of the resulting solution was determined at 670 nm with a spectrophotometer. The H_2_S concentration was calculated against the calibration curve of the standard H_2_S solution.

### Isolation of primary rat nucleus pulposus cells

NP cells were isolated from 4-week-old male Wistar rats (150–200 g) as previously described [1]. Rats were sacrificed with CO_2_, the spinal columns were removed en bloc under aseptic conditions and the lumbar intervertebral disc were collected. Using a dissecting microscope, the gel-like NP was separated from the fibrous annulus and digested with 0.1% type II collagenase (Sigma, St. Louis, MO) and 2 U/ml hyaluronidase (Sigma, St. Louis, MO) for 4 hours. The partially digested tissue was maintained as an explant in a humidified atmosphere containing 5% CO_2_ at 37°C in complete culture medium: Dulbecco’s Modified Eagle Media: Nutrient Mixture F-12 (DMEM/F12) supplemented with 10% fetal bovine serum (FBS) and antibiotics After the cultured cells reached confluence, trypsin (0.25%) /EDTA (1mmol/L) solution (Invitrogen, USA) was applied and cells were collected and sub-cultured. The third passage of cells were collected for following analysis.

Type II collagen and aggrecan in NP cells was detected by immunohistochemistry staining. Rat NP cells were fixed with 4% paraformaldehyde on ice for 20 minutes and permeabilized for 15 minutes with PBS containing 0.5% triton-X 100, then incubated in blocking solution (10% FBS in PBS) for 45 minutes. Primary antibody of rabbit anti-Type II collagen (1:100, Zhong shan jin qiao, CN) and mouse anti-aggrecan (1:100, Usbiological, USA) were applied overnight at 4°C. Cells incubated with mouse isotype IgG control were used as control. Cells were then incubated with FITC conjugated anti-mouse IgG (1:200, Zhong shan jin qiao, CN) for 1 hour followed with DAPI incubation for 20 minutes at room temperature. Staining pictures were obtained using laser scanning confocal microscope (Olympus Fluoview, Olympus Corp, JP). Ultra structure features were observed by transmission electron microscope. The study was approved by the Animal Ethics Committee of Peking University First Hospital.

### Immunofluorescence staining in rat nucleus pulposus cells

Rat NP cells were fixed with 4% paraformaldehyde on ice for 20 minutes and permeabilized for 15 minutes with PBS containing 0.5% triton-X 100 then incubated in blocking solution (10% FBS in PBS) for 45 minutes. Primary antibody of mouse anti-CBS and mouse anti-CSE (1:100, Santa Cruz, USA) were applied overnight at 4°C. Cells incubated with mouse isotype IgG control were used as control. After washing with PBS, cells were incubated with FITC conjugated anti-mouse IgG (1:200, Zhong shan jin qiao, CN) for 1 hour followed with DAPI incubation for 20 minutes at room temperature. Images were obtained using laser scanning confocal microscope (Olympus Fluoview, Olympus Corp, JP).

### Hypoxia in primary rat nucleus pulposus cells

Hypoxia in rat NP cells was induced using CoCl_2_ (150μmol/L) [16]. NaHS (150μmol/L) was used as exogenous H_2_S donor [17, 18]. The third passage of NP cells were used for analysis and were divided into 4 groups: 1) Control; 2) NaHS; 3) Hypoxia; 4) NaHS+Hypoxia. The cells were then cultured for 72 hours after the treatments.

### Annexin V—FITC staining detection

After treatments NP cells were incubated with 5ul of annexin V (Keygen, CN) for 15 minutes in the dark at room temperature. Cells were observed using fluorescence inverted phase contrast microscope (excitation 490nm, emission 520nm, DMI 60000, Leica, GE). Samples were also quantified with the inverted phase contrast lens of the same field to document all the cells in the image field. Each sample was evaluated by 2 investigators in blinded manner, and the percentage of annexin V positive cells in total cells was calculated.

### Western blot

NP cells were harvested in RIPA buffer (Keygenbio, China), and total protein concentration was quantified using the BCA protein assay kit (Keygenbio, China). Protein extracts were electrophoresized in 5–12% Bis–Tris gel, then transferred into polyvinylidene difluoride (PVDF) membranes. Membranes were blocked in 5% fat-free milk (Sigma, USA) for 1 hours at room temperature, and incubated with primary antibodies rabbit anti-Fas (1:500, Santa Cruz, USA), rabbit anti-Cytochromes C (CytC) (1:500, Santa Cruz, USA), rabbit anti-Caspase 9 (1:600, Santa Cruz, USA), rabbit anti-cleaved Caspase 3 (1:600, Santa Cruz, USA), and rabbit anti-GAPDH (1:5000, Santa Cruz, USA) at 4°C overnight with gentle agitation. The membranes were then incubated with horseradish peroxidase-conjugated goat anti-rabbit secondary antibody (1:8000, Santa Cruz, USA) for 1 hours at room temperature. Immunolabeling was detected using enhanced chemiluminescence reagent (Millipore, USA). Protein bands were quantified using densitometry and analyzed using image J software (vertion.1.42q, National Institutes of Health, US).

### Statistical analysis

Data were analyzed by Statistical package for program (Version12, SPSS, Chicago, IL), one-way ANOVA followed by Bonferroni post hoc test was used for comparison between groups. Data are presented as means ± SEM, statistical significance was defined as p<0.05.

## Results

### CBS and CSE expression in human lumbar nucleus pulposus

**Fig 1:**
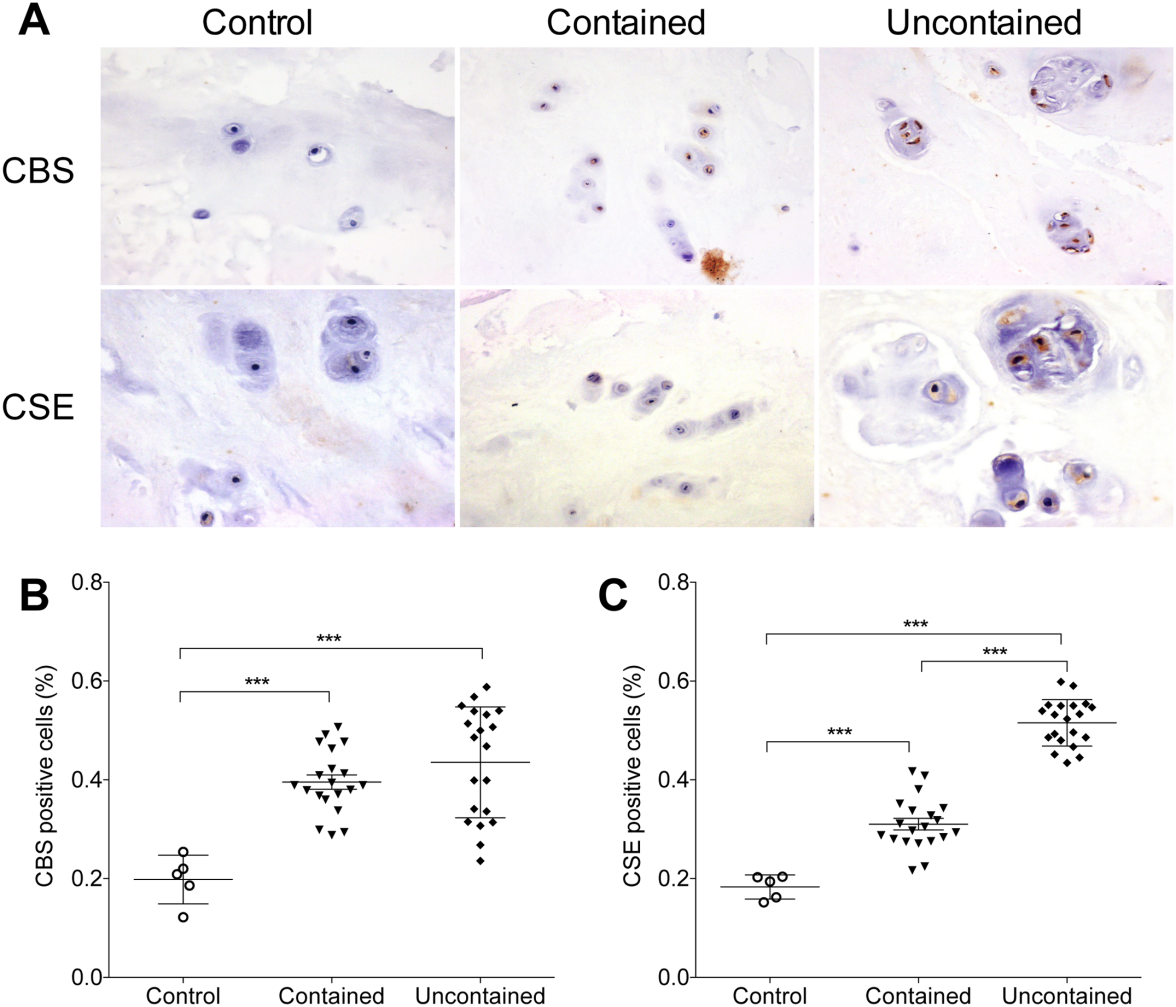
Immunohistochemistry staining of CBS and CSE in human nucleus pulposus tissue. **(A)** Representative images of CBS and CSE staining (×200). **(B, C)** CBS and CSE positive staining are significantly increased in NP cells in contained and uncontained herniation groups. In addition, NP cells from uncontained group show significant higher CBS and CSE expression compared to the contained group (*** p<0.001; n = 5 in control; n = 20 in contained and uncontained groups; CBS: cystathionine-β-synthase; CSE: cystathionine γ-lyase).

To determine whether endogenous H_2_S has impact on the intervertebral disc herniation, we detected the CBS and CSE expression in the NP cells isolated from herniated discs. The immunohistochemistry showed CBS and CSE positive staining are significantly increased in NP isolated from patients with contained and uncontained herniation (p<0.001 vs control respectively, Fig 1). Furthermore, cells from patients with uncontained herniation showed significant higher CSE expression (0.52 ± 0.01% CSE positive cells) compared to the contained herniation group (0.31 ± 0.01%, p<0.001, Fig 1A and 1C).

### H2S generation in human nucleus pulposus

**Fig 2:**
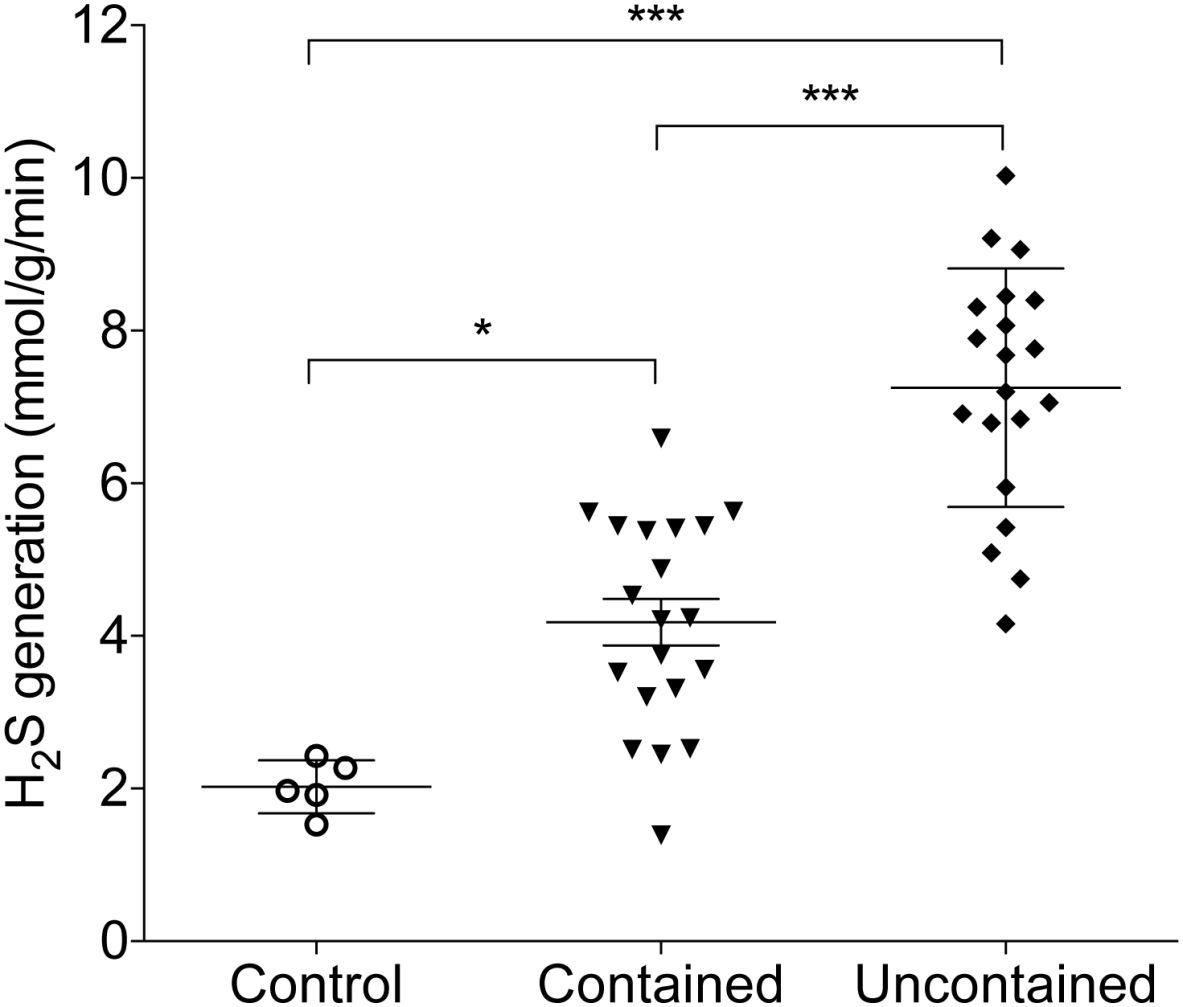
The H_2_S production in human nucleus pulposus tissue. The H_2_S production in human NP biopsy were detective using sulphur-sensitive electrode. There is significant increased H_2_S in both contained and uncontained herniation groups compared to control. Furthermore, NP cells isolated from patients in uncontained group show significant higher H_2_S production compared to contained group. (* p<0.05; *** p<0.001; n = 5 in control; n = 20 in contained and uncontained groups).

After determining the relationship between CBS, CSE and intervertebral disc herniation, we further evaluated the H_2_S generation from NP biopsy. The H_2_S production rates was significantly higher in both contained (4.18 ± 0.31 mmol/g/min) and uncontained herniation group (7.25 ± 0.35 mmol/g/min) compared to control (2.02 ± 0.16 mmol/g/min, p<0.05 and p<0.001 respectively). Furthermore, NP cells isolated from patients with uncontained herniation showed significant higher H_2_S production rate compared to contained group (p<0.001, Fig 2).

### Apoptosis in human nucleus pulposus

**Fig 3:**
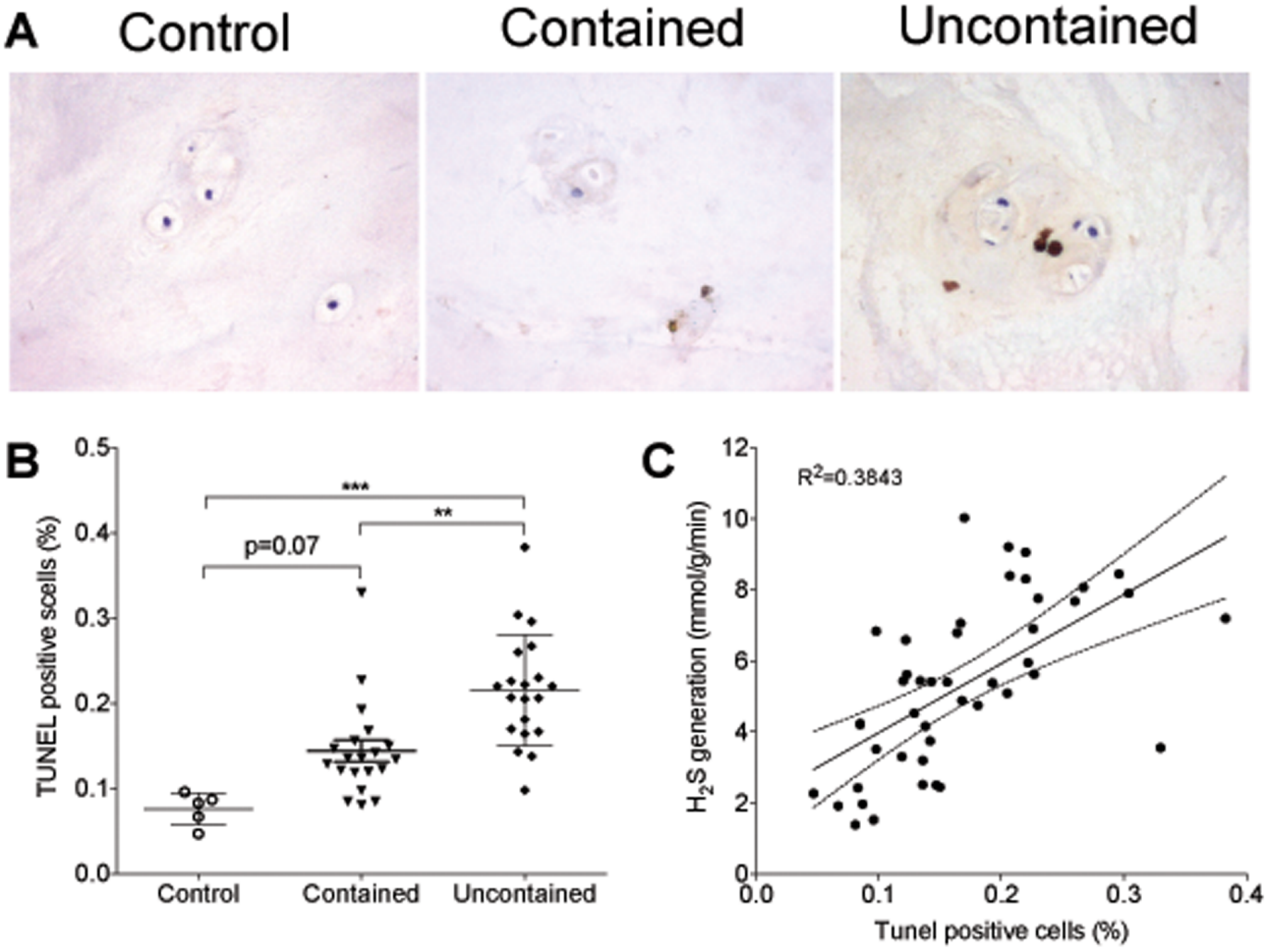
TUNEL staining in human nucleus pulposus tissue. **(A)** Representative images of TUNEL staining (x200). **(B)** The percentage of TUNEL positive cells are higher in contained herniation group and in uncontained herniation group. In addition, the proportion of apoptotic cells is significant higher in uncontained herniation group compared to the contained group. **(C)** A significant correlation is observed between H_2_S generation rate and cells apoptosis in NP samples (* p<0.05; ** p<0.01; *** p<0.001; n = 5 in control; n = 20 in contained and uncontained groups).

NP isolated from herniated disc contains increased proportion of apoptotic cells compared to control. The TUNEL positive cells were 0.14 ± 0.01% in contained herniation group and 0.22 ± 0.01% in uncontained herniation group (p = 0.07 and p<0.001 vs control respectively, Fig 3A and 3B). The proportion of apoptotic cells was significant higher in uncontained herniation group compared to the contained group (p<0.01, Fig 3A and 3B). In addition, there was significant correlation between H_2_S generation rate and cells apoptosis in NP samples (R^2^ = 0.3843, p<0.001, Fig 3C).

### Expression of CBS and CSE in rat nucleus pulposus cells

**Fig 4:**
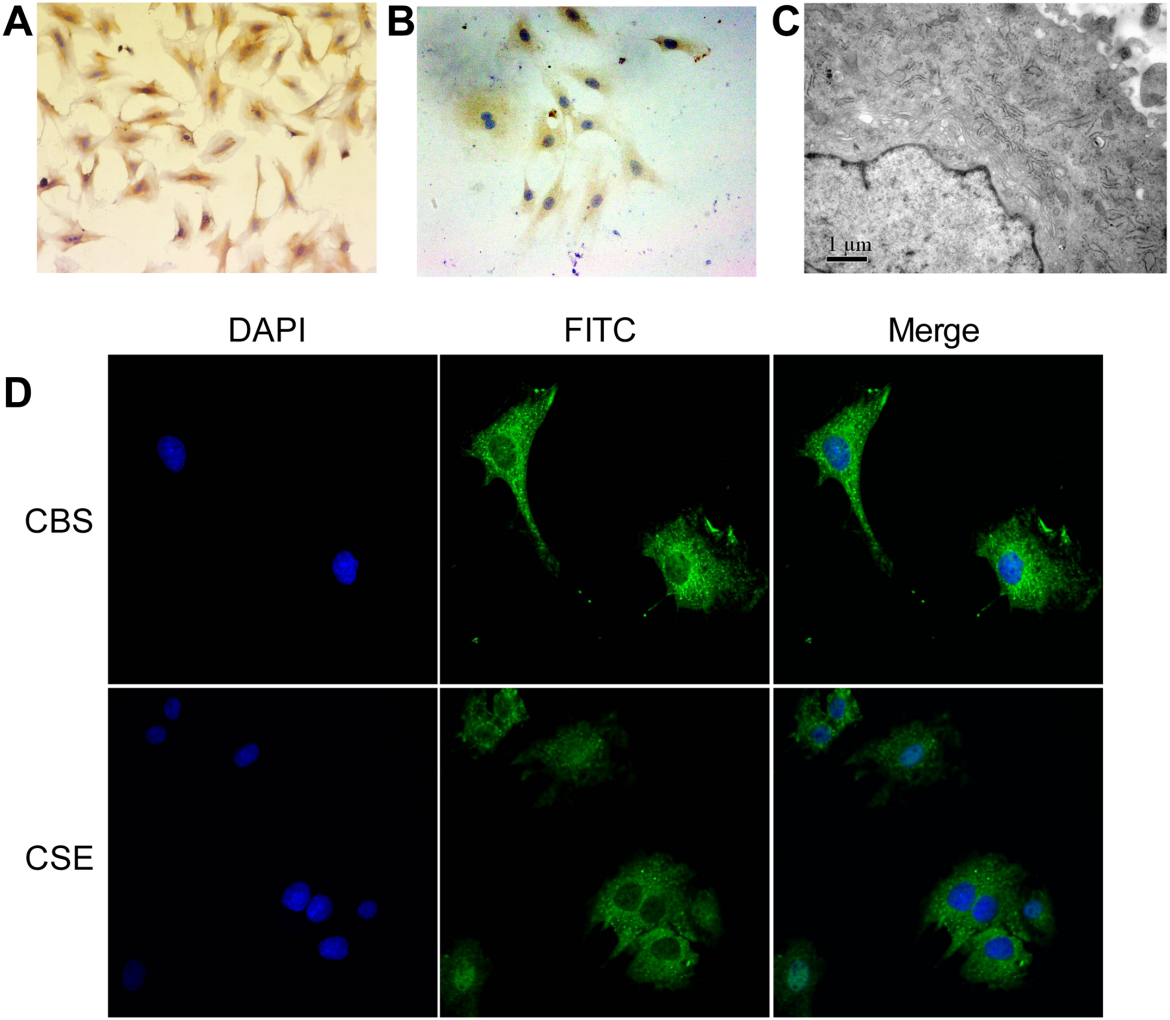
Identification of cultured rat NP cells and immunofluorescent detection of CBS and CSE in rat NP cells. Images indicate the third passage of rat NP cells are positive in type II colleagen **(A)** and aggrecan **(B)**. Transmission electron microscope show NP cells contain endoplasmic reticulum, Golgi complexes, free ribosomes and several multilamellar bodies **(C)**. Immunofluorescent staining showed CBS and CSE are expressed in the cytoplasm of the rat NP cells **(D)** (n = 6/group; CBS: cystathionine-β-synthase; CSE: cystathionine γ-lyase).

The third passage of rat NP cells appeared round or multi-angular under inverted phase contrast microscope. Cells that were positive in type II colleagen (Fig 4A) and aggrecan (Fig 4B) were identified as NP cells. Endoplasmic reticulums, Golgi complexes, free ribosomes and multilamellar bodies were observed under transmission electron microscope (Fig 4C).

Immunofluorescence staining showed both CBS and CSE were expressed in the cytoplasm of the rat NP cells (Fig 4D).

### H2S inhibits hypoxia induced apoptosis in rat nucleus pulposus cells

**Fig 5:**
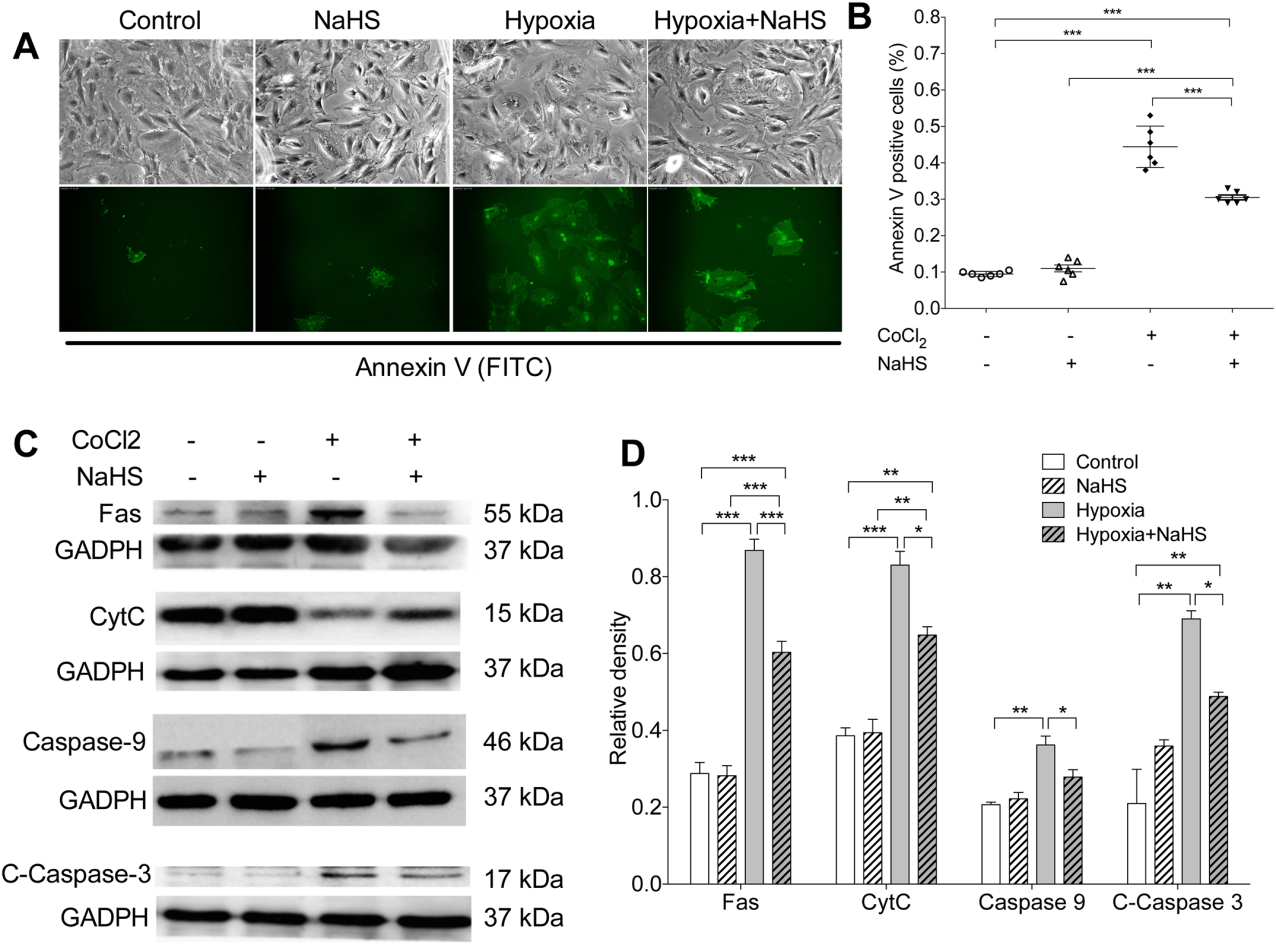
H_2_S inhibits hypoxia induced apoptosis in rat nucleus pulposus cells. **(A)** Representative images of cultured rat NP cells and corresponding Annexin V–FITC staining in each group. **(B)** There were significant higher apoptotic rates in the hypoxia group and the hypoxia+NaHS group than the control group; but lower apoptotic rate in the hypoxia +NaHS group than the hypoxia group. **(C, D)** Hypoxia significantly increased the protein levels of Fas, CytC, Caspase 9 and cleaved-Caspase-3 in both NaHS treated and non-treated NP cells. These increases were significantly eliminated by administration of NaHS (* p<0.05; ** p<0.01; *** p<0.001; n = 6/group in A & B; n = 3/group in C & D; CytC: cytochrome C).

The Annexin V staining showed significant increased apoptosis in primary rat NP cells after CoCl_2_ induced hypoxia (p<0.001 vs control, Fig 5A and 5B), which was prevented by simultaneous exogenous NaHS treatment (p<0.001, Fig 5A and 5B). The NaHS alone did not affect the apoptosis in rat NP cells.

To further investigate the mechanisms how H_2_S affects hypoxia induced apoptosis in NP cells, we determined multiple proteins associated with Fas-mediated pathway which plays pivotal role in regulating apoptosis. Hypoxia significantly increased the protein levels of Fas, CytC, Caspase 9 and cleaved-Caspase-3 in both NaHS treated and non-treated NP cells. These increases were significantly eliminated by the administration of NaHS (Fig 5C and 5D).

## Discussion

The intervertebral disc is the largest avascular structure in body. Within intervertebral disc, NP cells consume glucose to generate energy thus there is a small but significant consumption of oxygen [19]. Aging and cumulative damages on intervertebral disc lead to degenerative changes in disc, which coincide with reduction in oxygen and other nutrition supply. In this manuscript, we document the endogenous generation of H_2_S in human intervertebral disc and demonstrate this endogenous H_2_S generation rate is associated with NP cells apoptosis. Using primary rat NP cells, we show that the hypoxia induced apoptosis can be attenuated by exogenous H_2_S donor.

The role of NO in regulating cell apoptosis in intervertebral disc and in disc degeneration have been well discussed [20, 21]. H_2_S is another pivotal gasotransmitter and has similar biological effects as NO in multiple diseases scenarios. Studies have revealed the impotant role of H_2_S in the degenerative diseases such as Alzheimer’s disease, Parkinson’s disease and retinal degeneration [22–24]. One of the proposed mechanism is that H_2_S protects cells from hypoxia induced apoptosis [7].

In this study, we utilized the biopsy from patients with disc herniation and for the first time, proved the presence of H_2_S in the NP tissue using sulphur-sensitive electrode, which is a well-established method to specifically detect H_2_S production in tissue [14, 15]. In addition, we found two key enzymes of H_2_S, CBS and CSE, were also expressed in human NP tissue. Thus, both direct and indirect methods indicated the presence of H_2_S in human intervertebral disc.

The severity of disc herniation accompanies with different hypoxic conditions [3]. We validated that the disc herniation is associated with NP cell apoptosis, and the percentage of apoptotic cells is significantly linked to the severity of herniation. Interestingly, the H_2_S generation rate also shows significant positive correlation with cells apoptosis in NP biopsy. These data indicated that H_2_S may play pivotal role in regulating NP cells apoptosis in hypoxic environment. In addition, we also observed that the expression of CBS and CSE in NP biopsy is associated with the severity of the disc herniation. Study from Xu *et al* also demonstrated the protective effects of H_2_S against NP cells apoptosis and intervertebral disc degeneration [12]. Interestingly, the authors found advanced disc degeneration was accompanied with reduced CBS and CSE expression. We should point out that our work evaluated the relationship between CBS/CSE expression and the severity of disc herniation from patients at similar ages. However, Xu’s study compared the CBS/CSE expression in discs with different degeneration grades, while the patients were at different ages and with different udisc diseases.

To further validate the potential biological function of H_2_S in NP cells apoptosis, we cultured primary rat NP cells and used CoCl_2_ to mimic hypoxic condition. To avoid aging and dedifferentiation of NP cells during monolayer culture [25, 26], we only used the third passage of rat NP cells for analysis. We also verified the NP cells by positive type II collagen and aggrecan staining [27, 28].

We then identified the cytoplasmic expression of CBS and CSE in the NP cells. This further validates the presence of endogenous H_2_S in the cultured rat NP cells. Accumulating evidences showed that H_2_S effectively modulates cell apoptosis in different systems and this function is associated with hypoxic environment [6, 7]. NaHS, a potent H_2_S donor, can attenuate the death of myocardiocytes under hypoxia [29]. In our study, we found the CoCl_2_ induced NP cells apoptosis was significantly eliminated by NaHS. In addition, NaHS significantly attenuated the increase of Fas, CytC, Caspase-9 and cleaved Caspase-3 in NP cells following hypoxia. Fas signaling plays critical role in regulating p53-dependent apoptosis during hypoxia [30]. The release of CytC from mitochondria is a pivotal initiator for caspase activation, and subsequently mediates cell death [31]. Thus, our data suggested the NaHS regulates NP cells apoptosis via Fas and CytC dependent pathways. This may also be an important mechanism that H_2_S regulating the degenerative changes in the intervertebral discs during aging and diseases. The underlying signaling pathways of H_2_S regulating programed cell death in intervertebral disc is a critical question and requires further investigation.

Taking together, our study demonstrated that human NP tissue generates endogenous H_2_S, and exogenous H_2_S donor prevents the hypoxia induced NP cell apoptosis *in vitro*. Understanding the mechanisms of how H_2_S modulates the program death of NP cells may provide insights in developing novel strategies for treating and preventing degenerative disc diseases.

## Supporting information

S1 Dataset Data of Figs 1 and 3. (XLS)

S2 Dataset Data of Fig 2. (XLS)

S3 Dataset Data of Fig 5B. (XLS)

S4 Dataset Data of Fig 5D. (XLSX)

## References

[1] Andersson GB. Epidemiological features of chronic low-back pain. Lancet. 1999;354(9178):581–5. doi: 10.1016/S0140-6736(99)01312-4 . 10470716 10.1016/S0140-6736(99)01312-4

[2] Wang HQ, Yu XD, Liu ZH, Cheng X, Samartzis D, Jia LT, et al Deregulated miR-155 promotes Fas-mediated apoptosis in human intervertebral disc degeneration by targeting FADD and caspase-3. J Pathol. 2011;225(2):232–42. doi: 10.1002/path.2931 . 21706480 10.1002/path.2931

[3] Ha KY, Koh IJ, Kirpalani PA, Kim YY, Cho YK, Khang GS, et al The expression of hypoxia inducible factor-1alpha and apoptosis in herniated discs. Spine (Phila Pa 1976). 2006;31(12):1309–13. doi: 10.1097/01.brs.0000219493.76081.d6 . 16721291 10.1097/01.brs.0000219493.76081.d6

[4] Risbud MV, Guttapalli A, Albert TJ, Shapiro IM. Hypoxia activates MAPK activity in rat nucleus pulposus cells: regulation of integrin expression and cell survival. Spine (Phila Pa 1976). 2005;30(22):2503–9. . 16284587 10.1097/01.brs.0000186326.82747.13

[5] Farrugia G, Szurszewski JH. Carbon monoxide, hydrogen sulfide, and nitric oxide as signaling molecules in the gastrointestinal tract. Gastroenterology. 2014;147(2):303–13. doi: 10.1053/j.gastro.2014.04.041 . 24798417 10.1053/j.gastro.2014.04.041 PMC4106980

[6] Chunyu Z, Junbao D, Dingfang B, Hui Y, Xiuying T, Chaoshu T. The regulatory effect of hydrogen sulfide on hypoxic pulmonary hypertension in rats. Biochem Biophys Res Commun. 2003;302(4):810–6. . 12646242 10.1016/s0006-291x(03)00256-0

[7] Luo Y, Liu X, Zheng Q, Wan X, Ouyang S, Yin Y, et al Hydrogen sulfide prevents hypoxia-induced apoptosis via inhibition of an H2O2-activated calcium signaling pathway in mouse hippocampal neurons. Biochem Biophys Res Commun. 2012;425(2):473–7. doi: 10.1016/j.bbrc.2012.07.131 . 22846576 10.1016/j.bbrc.2012.07.131

[8] Zhang Y, Tang ZH, Ren Z, Qu SL, Liu MH, Liu LS, et al Hydrogen sulfide, the next potent preventive and therapeutic agent in aging and age-associated diseases. Mol Cell Biol. 2013;33(6):1104–13. doi: 10.1128/MCB.01215-12 . 23297346 10.1128/MCB.01215-12 PMC3592015

[9] Peter EA, Shen X, Shah SH, Pardue S, Glawe JD, Zhang WW, et al Plasma free H2S levels are elevated in patients with cardiovascular disease. J Am Heart Assoc. 2013;2(5):e000387 Epub 2013/10/25. doi: 10.1161/JAHA.113.000387 . 24152982 10.1161/JAHA.113.000387 PMC3835249

[10] Aminzadeh MA, Vaziri ND. Downregulation of the renal and hepatic hydrogen sulfide (H2S)-producing enzymes and capacity in chronic kidney disease. Nephrol Dial Transplant. 2012;27(2):498–504. Epub 2011/11/01. doi: 10.1093/ndt/gfr560 . 22036943 10.1093/ndt/gfr560

[11] di Masi A, Ascenzi P. H2S: a "double face" molecule in health and disease. Biofactors. 2013;39(2):186–96. Epub 2012/12/13. doi: 10.1002/biof.1061 . 23233276 10.1002/biof.1061

[12] Xu D, Jin H, Wen J, Chen J, Chen D, Cai N, et al Hydrogen sulfide protects against endoplasmic reticulum stress and mitochondrial injury in nucleus pulposus cells and ameliorates intervertebral disc degeneration. Pharmacol Res. 2017;117:357–69. Epub 2017/01/15. doi: 10.1016/j.phrs.2017.01.005 . 28087442 10.1016/j.phrs.2017.01.005

[13] Yang T, Zhuang L, Rei Fidalgo AM, Petrides E, Terrando N, Wu X, et al Xenon and sevoflurane provide analgesia during labor and fetal brain protection in a perinatal rat model of hypoxia-ischemia. PLoS One. 2012;7(5):e37020 doi: 10.1371/journal.pone.0037020 . 22615878 10.1371/journal.pone.0037020 PMC3355162

[14] Li W, Tang C, Jin H, Du J. Regulatory effects of sulfur dioxide on the development of atherosclerotic lesions and vascular hydrogen sulfide in atherosclerotic rats. Atherosclerosis. 2011;215(2):323–30. doi: 10.1016/j.atherosclerosis.2010.12.037 . 21300352 10.1016/j.atherosclerosis.2010.12.037

[15] Yang J, Li H, Ochs T, Zhao J, Zhang Q, Du S, et al Erythrocytic hydrogen sulfide production is increased in children with vasovagal syncope. J Pediatr. 2015;166(4):965–9. Epub 2015/02/03. doi: 10.1016/j.jpeds.2014.12.021 . 25641243 10.1016/j.jpeds.2014.12.021

[16] Torii S, Goto Y, Ishizawa T, Hoshi H, Goryo K, Yasumoto K, et al Pro-apoptotic activity of inhibitory PAS domain protein (IPAS), a negative regulator of HIF-1, through binding to pro-survival Bcl-2 family proteins. Cell Death Differ. 2011;18(11):1711–25. doi: 10.1038/cdd.2011.47 . 21546903 10.1038/cdd.2011.47 PMC3190112

[17] Pan TT, Feng ZN, Lee SW, Moore PK, Bian JS. Endogenous hydrogen sulfide contributes to the cardioprotection by metabolic inhibition preconditioning in the rat ventricular myocytes. J Mol Cell Cardiol. 2006;40(1):119–30. doi: 10.1016/j.yjmcc.2005.10.003 . 16325198 10.1016/j.yjmcc.2005.10.003

[18] Srilatha B, Adaikan PG, Li L, Moore PK. Hydrogen sulphide: a novel endogenous gasotransmitter facilitates erectile function. J Sex Med. 2007;4(5):1304–11. doi: 10.1111/j.1743-6109.2007.00561.x . 17655658 10.1111/j.1743-6109.2007.00561.x

[19] Liu J, Wang J, Zhou Y. Upregulation of BNIP3 and translocation to mitochondria in nutrition deprivation induced apoptosis in nucleus pulposus cells. Joint Bone Spine. 2012;79(2):186–91. doi: 10.1016/j.jbspin.2011.04.011 . 21676641 10.1016/j.jbspin.2011.04.011

[20] Kohyama K, Saura R, Doita M, Mizuno K. Intervertebral disc cell apoptosis by nitric oxide: biological understanding of intervertebral disc degeneration. Kobe J Med Sci. 2000;46(6):283–95. . 11501016

[21] Niu CC, Lin SS, Yuan LJ, Chen LH, Wang IC, Tsai TT, et al Hyperbaric oxygen treatment suppresses MAPK signaling and mitochondrial apoptotic pathway in degenerated human intervertebral disc cells. J Orthop Res. 2013;31(2):204–9. doi: 10.1002/jor.22209 . 22886767 10.1002/jor.22209

[22] Kida K, Yamada M, Tokuda K, Marutani E, Kakinohana M, Kaneki M, et al Inhaled hydrogen sulfide prevents neurodegeneration and movement disorder in a mouse model of Parkinson’s disease. Antioxid Redox Signal. 2011;15(2):343–52. doi: 10.1089/ars.2010.3671 . 21050138 10.1089/ars.2010.3671 PMC3118610

[23] Mikami Y, Shibuya N, Kimura Y, Nagahara N, Yamada M, Kimura H. Hydrogen sulfide protects the retina from light-induced degeneration by the modulation of Ca2+ influx. J Biol Chem. 2011;286(45):39379–86. doi: 10.1074/jbc.M111.298208 . 21937432 10.1074/jbc.M111.298208 PMC3234762

[24] Zhang H, Gao Y, Zhao F, Dai Z, Meng T, Tu S, et al Hydrogen sulfide reduces mRNA and protein levels of beta-site amyloid precursor protein cleaving enzyme 1 in PC12 cells. Neurochem Int. 2011;58(2):169–75. doi: 10.1016/j.neuint.2010.11.010 . 21095213 10.1016/j.neuint.2010.11.010

[25] He F, Pei M. Rejuvenation of nucleus pulposus cells using extracellular matrix deposited by synovium-derived stem cells. Spine (Phila Pa 1976). 2012;37(6):459–69. doi: 10.1097/BRS.0b013e31821fcc64 . 21540772 10.1097/BRS.0b013e31821fcc64

[26] Tsai TT, Guttapalli A, Oguz E, Chen LH, Vaccaro AR, Albert TJ, et al Fibroblast growth factor-2 maintains the differentiation potential of nucleus pulposus cells in vitro: implications for cell-based transplantation therapy. Spine (Phila Pa 1976). 2007;32(5):495–502. doi: 10.1097/01.brs.0000257341.88880.f1 . 17334282 10.1097/01.brs.0000257341.88880.f1

[27] Gan JC, Ducheyne P, Vresilovic EJ, Shapiro IM. Intervertebral disc tissue engineering II: cultures of nucleus pulposus cells. Clin Orthop Relat Res. 2003;(411):315–24. doi: 10.1097/01.blo.0000063797.98363.d3 . 12782890 10.1097/01.blo.0000063797.98363.d3

[28] Kluba T, Niemeyer T, Gaissmaier C, Grunder T. Human anulus fibrosis and nucleus pulposus cells of the intervertebral disc: effect of degeneration and culture system on cell phenotype. Spine (Phila Pa 1976). 2005;30(24):2743–8. . 16371897 10.1097/01.brs.0000192204.89160.6d

[29] Zhu YZ, Wang ZJ, Ho P, Loke YY, Zhu YC, Huang SH, et al Hydrogen sulfide and its possible roles in myocardial ischemia in experimental rats. J Appl Physiol (1985). 2007;102(1):261–8. doi: 10.1152/japplphysiol.00096.2006 . 17038495 10.1152/japplphysiol.00096.2006

[30] Liu T, Laurell C, Selivanova G, Lundeberg J, Nilsson P, Wiman KG. Hypoxia induces p53-dependent transactivation and Fas/CD95-dependent apoptosis. Cell Death Differ. 2007;14(3):411–21. Epub 2006/08/19. doi: 10.1038/sj.cdd.4402022 . 16917513 10.1038/sj.cdd.4402022

[31] Jiang X, Wang X. Cytochrome C-mediated apoptosis. Annu Rev Biochem. 2004;73:87–106. Epub 2004/06/11. doi: 10.1146/annurev.biochem.73.011303.073706 . 15189137 10.1146/annurev.biochem.73.011303.073706

